# A preliminary evaluation of efficacy and safety of Wharton’s jelly mesenchymal stem cell transplantation in patients with type 2 diabetes mellitus

**DOI:** 10.1186/scrt446

**Published:** 2014-04-23

**Authors:** Xuebin Liu, Pei Zheng, Xiaodong Wang, Guanghui Dai, Hongbin Cheng, Zan Zhang, Rongrong Hua, Xinxin Niu, Jing Shi, Yihua An

**Affiliations:** 1 Department of Cell Transplantation, General Hospital of Chinese people’s Armed Police Forces, Beijing 100039, China

## Abstract

**Introduction:**

Stem cell therapy has recently been introduced to treat patients with type 2 diabetes mellitus (T2DM). However, no data are available on the efficacy and safety of allogeneic Wharton’s Jelly-derived mesenchymal stem cell (WJ-MSC) transplantation in patients with T2DM. Here we performed a non-placebo controlled prospective phase I/II study to determine efficacy and safety of WJ-MSC transplantation in T2DM.

**Methods:**

Twenty-two patients with T2DM were enrolled and received WJ-MSC transplantation through one intravenous injection and one intrapancreatic endovascular injection (catheterization). They were followed up for 12 months after transplantation. The primary endpoints were changes in the levels of glycated hemoglobin and C-peptide and the secondary endpoints included insulin dosage, fasting blood glucose (FBG), post-meal blood glucose (PBG), inflammatory markers and T lymphocyte counts.

**Results:**

WJ-MSC transplantation significantly decreased the levels of glucose and glycated hemoglobin, improved C-peptide levels and beta cell function, and reduced markers of systemic inflammation and T lymphocyte counts. No major WJ-MSC transplantation-related adverse events occurred, but data suggest a temporary decrease in levels of C-peptide and beta cell function at one month after treatment, possibly related to intrapancreatic endovascular injection.

**Conclusions:**

Our data demonstrate that treatment with WJ-MSCs can improve metabolic control and beta cell function in patients with T2DM. The therapeutic mechanism may involve improvements in systemic inflammation and/or immunological regulation.

**Trial registration:**

Chinese Clinical Trial Register ChiCTR-ONC-10000985. Registered 23 September 2010

## Introduction

Type 2 diabetes mellitus (T2DM) is a metabolic stress resulting from over-nutrition- and insufficient activity-induced insulin resistance and β-cell impairment [1,2]. The continuing hyperglycemia results in both microvascular and macrovascular complications. It is important for patients to maintain nearly normal glycemic levels to reduce their risk of diabetic complications.

Although diet control, physical exercise and oral anti-diabetic drugs are all effective in decreasing hyperglycemia, it is difficult for many patients to achieve good glycemic control depending only on these options, and most of these patients will eventually require insulin therapy [3]. However, insulin treatment negatively impacts patients’ daily lives and is frequently associated with hypoglycemic episodes. Therefore, it is imperative to explore new strategies for optimal glycemic control or β-cell replacement.

In recent years, several animal studies and clinical trials have shown that mesenchymal stem cell (MSC) transplantation can improve glycemic control and beta cell function [4,5]. XY Li and colleagues designed a clinical study to treat foot disease in patients with type 2 diabetes mellitus using human umbilical cord blood mesenchymal stem cells (hUCB-MSC) and indicated that levels of blood glucose and required insulin dosage were reduced after hUCB-MSC transplantation accompanied by improved clinical profiles in diabetic patients [6]. However, the exact mechanisms of reversing hyperglycemia remain unknown. A chronic inflammatory process has been demonstrated in insulin-sensitive tissues and pancreatic islets, which results in insulin resistance and beta-cell destruction [7,8]. MSCs have demonstrated anti-inflammatory roles in the treatment of many diseases, such as myocardial infarction [9], lung injury [10] and systemic lupus erythematosus [11]. In addition, MSCs play a role in immunoregulation in the therapy of graft-versus-host disease [12] and autoimmune disorders [13]. Therefore, we hypothesized that Wharton’s Jelly mesenchymal stem cell (WJ-MSC) transplantation could be a therapeutic option in T2DM, and the mechanism may involve improvements in inflammation and immunoregulation.

On the basis of these observations, we initiated a prospective phase I/II study using WJ-MSCs in patients with T2DM. In this report, we explored the efficacy and safety of WJ-MSC transplantation in T2DM patients and followed up with them for 12 months after treatment.

## Methods

### Patients

T2DM patients who were diagnosed according to American Diabetes Association criteria [14] were eligible for participation. The inclusion criteria included the following: the patients were between the ages of 18 and 70 years, male or female; they had poor glycemic control with recent anti-diabetic therapies, including drugs and/or insulin injection for at least three months; they had a negative result in testing for the glutamic acid decarboxylase antibody; they had not been pregnant or nursing; they had a fasting blood glucose (FBG) level ≥7.0 mmol/L and HbA1c ≥7%; and they had a good organic sufficiency, including heart, liver, kidney and lung, to receive interventional therapy. The exclusion criteria included the following: acute or chronic infections; any malignancies; hematological diseases or coagulopathy; known immunosuppressive disease (for example, acquired immunodeficiency); acute or chronic pancreatitis; and a history of thoracic or abdominal aorta diseases. The study protocol was approved by the Committees of Ethics in Research of the General Hospital of Chinese People’s Armed Police Forces. All of the patients provided written informed consent and confirmed their willingness to receive WJ-MSC injection and perform glucose self-monitoring.

### Study design

This single-center prospective phase I/II study involved 23 T2DM patients who were enrolled from 1 May 2010 to 1 May 2011. The patients received transplantation twice. During and after the treatment, the patients maintained their baseline anti-diabetic therapy, dietary habits and other lifestyle habits. The study had a follow-up period of 12 months for all of the patients after their last implantation.

### Preparation of human Wharton’s Jelly mesenchymal stem cells

**Figure 1:**
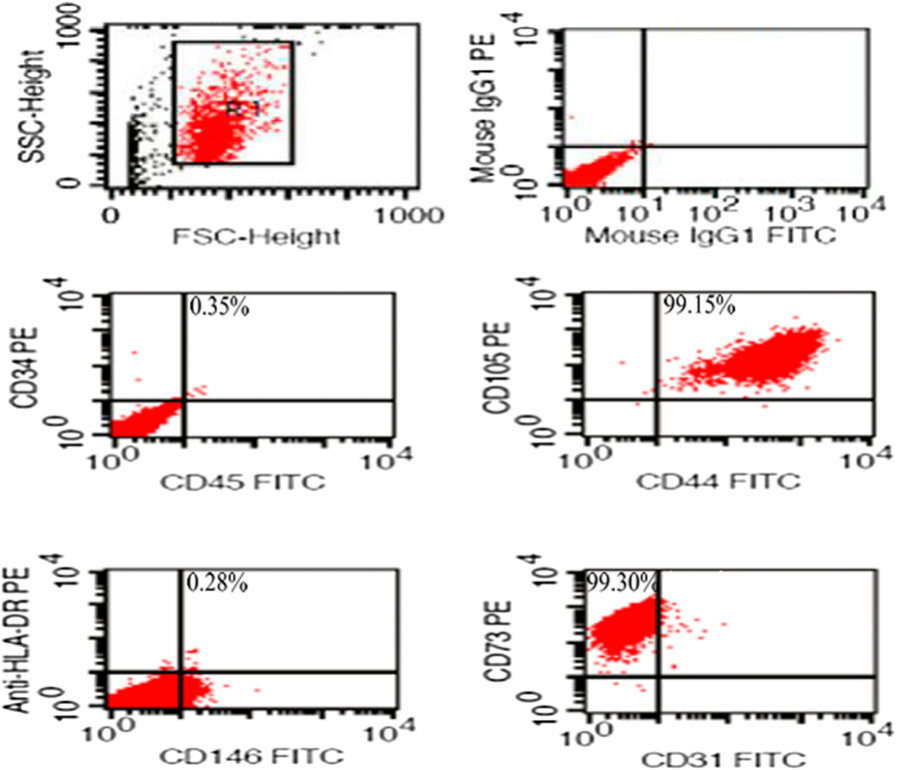
**Fluorescence-activated cell sorting analysis of umbilical cord mesenchymal stem cells.** Percentages of all CD105, CD73 and CD44 were higher than 95%, while none of CD34, CD45, CD31, CD146 and HLA-DR’s percentage was higher than 1%. WJ-MSCs, Wharton’s Jelly mesenchymal stem cells.

With the written consent of the parents, fresh human umbilical cords from both sexes were collected. All umbilical cords were obtained from healthy term fetus and were negative for hepatitis B virus (HBV), hepatitis C virus (HCV), human immunodeficiency virus (HIV), Epstein-Barr virus (EBV), cytomegalovirus (CMV) and syphilis in umbilical cord blood serum. After being disinfected in 75% ethanol for 30 seconds, the blood vessels were removed. WJ-MSCs were prepared as described elsewhere [11] and all culture conditions adhere to current Good Manufacturing Practice (cGMP) standards. Briefly, the cord was cut into cubes of approximately 0.5 cm^3^ and centrifuged at 250 g for five minutes. Following removal of the supernatant fraction, these cubes were washed with serum-free Dulbecco’s modified Eagle’s medium (DMEM) (Gibco Invitrogen, Carlsbad, CA, USA) and centrifuged at 250 g for five minutes. After aspiration of the supernatant fraction, the cubes were placed in a six-well plate (Corning Enterprises, Corning, NY, USA), cultured in DMEM supplemented with 10% FBS (StemCell Technologies, Vancouver, BC, Canada) (tested for safety in the same way as the umbilical cord blood serum), and 100 units/mL penicillin/streptomycin, and incubated at 37°C in a humidified tissue culture incubator containing 5% CO_2_ and 95% air, with a change of culture medium every three to five days. After 10 days in culture, the cord cubes were removed from culture and the adherent cells from individual explanted cord tissue were trypsinized and passaged into a new flask for further expansion. The WJ-MSCs harvested from passage 3 were frozen before characterizing MSC markers with flow cytometry. Flow cytometry results showed that ≥95% of cells expressed CD105, CD73, CD44, while the expression of CD45, CD34, CD31, CD146 and HLA-DR was 2% or less (Figure 1). One week before transplantation, the cryopreserved WJ-MSCs were thawed for further culture and expansion. WJ-MSCs between passages 4 and 6 were used for transplantation after characterizing MSC markers with flow cytometry. WJ-MSCs were infused with normal saline by vein (100 ml) and intervention (15 ml).

### Wharton’s Jelly mesenchymal stem cell transplantation

All of the patients were hospitalized and received WJ-MSC transplantation twice. The cells were infused into the peripheral vein on Day 5 the first time and were directly delivered to the pancreas via the splenic artery using endovascular catheters on Day 10 the second time. Each time, the number of cells used was 1 × 10^6^ per kilogram of body weight.

### Biochemical and clinical measurements

All of the tests were performed in the Laboratory Department of the General Hospital of Chinese People’s Armed Police Forces, Beijing, China. The glucose concentration was determined with the glucose oxidase method, glycated hemoglobin levels were measured using high-performance liquid chromatography (HPLC) and serum C-peptide levels were measured with a radioimmunoassay using commercial kits (Bio-Ekon Biotechnology Co., Ltd., Beijing, China). The patients were asked to forgo their anti-diabetic medication and to fast for eight hours before the test. The levels of cytokines (IL-1β, IL-6, IL-10 and TNF-α) in the serum were measured at baseline and at month 6 after WJ-MSC transplantation using the BD Cytometric Bead Array (CBA) Human Inflammatory Cytokines Kit according to the manufacturer's recommendations (BD Biosciences, Franklin, NJ, USA). Peripheral blood T lymphocyte subset counts were performed at baseline and six months after WJ-MSC transplantation with the Human Lymphocyte Subgroup Typing Kit according to the manufacturer's instructions (BD Biosciences).

### Study endpoints

The primary endpoints were changes in the glycated hemoglobin and C-peptide levels during the oral glucose tolerance test and at 1, 3, 6 and 12 months following WJ-MSC transplantation. The secondary endpoints included changes in the oral anti-diabetic drugs and/or insulin dosage; changes in FBG and 2 h post-meal blood glucose (PBG) levels; a change in β cell function, as assessed with the homeostasis model assessment (HOMA) calculator [15] with fasting glucose and C-peptide [16]; changes in the serum levels of IL-1β, IL-6, IL-10 and TNF-α as markers of systemic inflammation; and changes in peripheral blood T lymphocyte counts.

### Statistical analysis

All of the data are shown as the mean ± SD. Comparisons of time-dependent changes at the time of baseline and at 1, 3, 6 and 12 months after the treatment were performed using the repeated-measures ANOVA and *post hoc* analysis with the Bonferroni correction was applied. Correlation analyses were performed using Pearson’s test. A significant difference was indicated by *P* <0.05. All statistical analyses were performed using SPSS (Version 13.0 for Windows, SPSS, Chicago, IL, USA).

## Results

### Patients

From 1 May 2010 to 1 May 2011, we recruited 23 patients (16 male and 7 female); 1 male patient quit the study for unexplained reasons. The patients’ mean (±SD) age was 52.9 ± 10.5 years, and their mean disease duration was 8.7 ± 4.3 years. Their baseline demographic and biochemical parameters are summarized in Table 1. Of the included 22 patients, 17 patients received exogenous insulin infusion, with an average dose of 0.49 ± 0.22 IU/kg/day and a maximum of 1.2 IU/kg/day; 7 of these 17 patients also received oral anti-diabetic drugs at the same time. The other patients received only oral hypoglycemic agents (metformin or acarbose). All of the patients underwent WJ-MSC transplantation at our hospital. The patients received no other treatments in addition to their routine diabetic regimen, and they maintained their regular diets and lifestyle habits during their hospitalization and follow-up. All of the patients were followed up for 12 months.

**Table 1:** Baseline characteristics of the patients

Age (yrs)	52.9 ± 10.5*
Sex (no.)	
Male	15
Female	7
Body-mass index (kg/m^2^)*	25.1 ± 2.4
Weight (kg)	70.5 ± 11.7
Glycated hemoglobin (%)	8.20 ± 1.69
Fasting plasma glucose (mmol/L)	7.53 ± 2.67
Fasting C-peptide (ng/mL)	1.29 ± 0.83
Diabetes duration (yrs)	8.7 ± 4.3
Smoking status (%)	4
Cholesterol (mmol/L)	
Total	5.08 ± 0.84
HDL	1.08 ± 0.21
LDL	3.13 ± 0.72
TG	2.01 ± 1.63
Creatinine (μmol/L)	61.97 ± 19.93
Blood pressure (mm Hg)	
Systolic	134.5 ± 10.3
Diastolic	85.75 ± 6.56
Use of antihypertensive medication (%)	4
History of cardiovascular disease (%)	2
Albumin excretion rate >30 mg/L/24 hr (%)	4
Retinopathy (%)	11
Antidiabetic treatment (%)	
Oral drug	2
Insulin (alone or with oral drug)	17

*Plus/minus values are means ± SD. The body mass index is the weight in kilograms divided by the square of the height in meters. BMI*,* body mass index; HDL*,* high-density lipoprotein; LDL*,* low-density lipoprotein; TG*,* total cholesterol.

### Glycated hemoglobin

**Figure 2:**
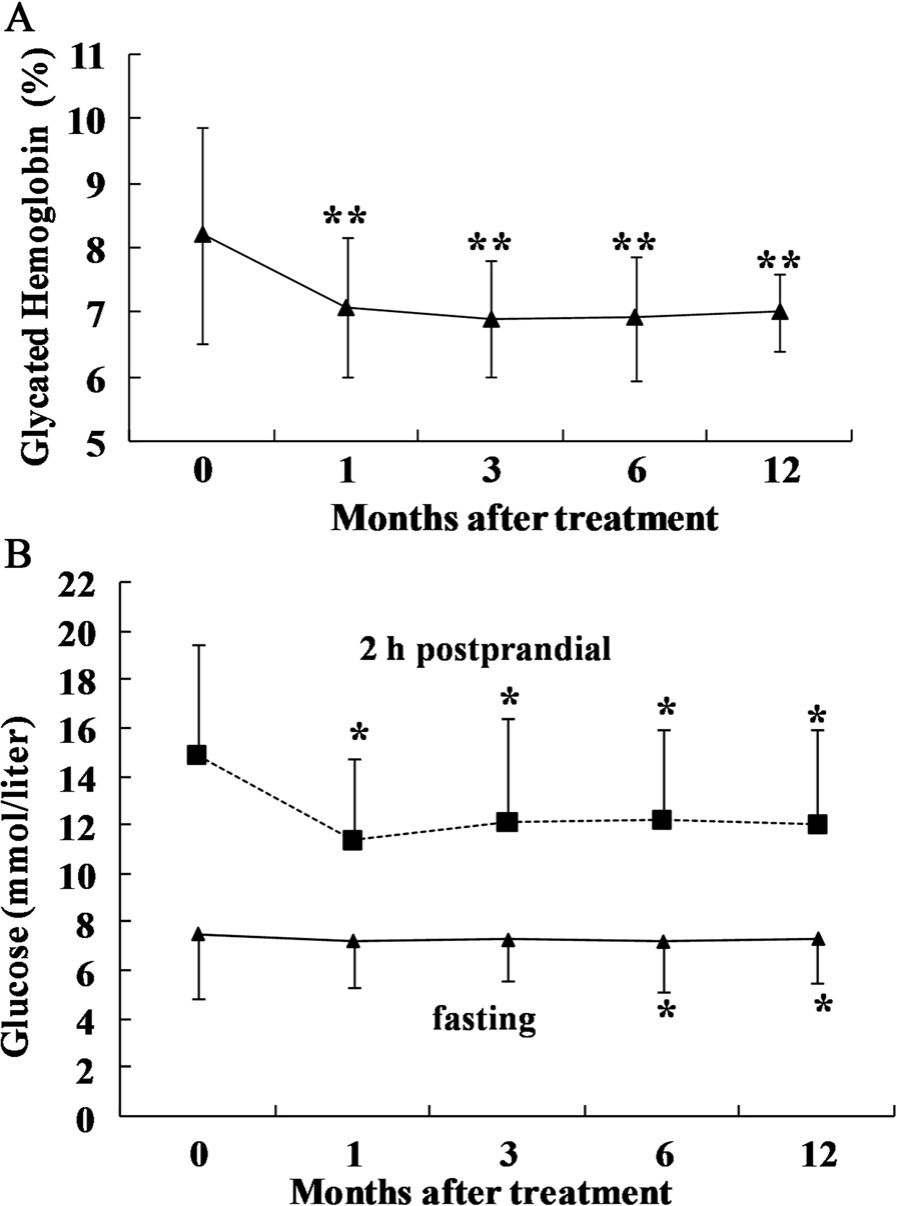
**Changes in glycated hemoglobin, fasting glucose and 2 h postprandial glucose levels during the 12-month study period. A**: The changes in glycated hemoglobin levels between baseline and 1, 3, 6 and 12 months. **B**: Changes in fasting glucose and 2 h postprandial glucose levels between baseline and 1, 3, 6 and 12 months. The results are shown as the mean ± S.D. *P <0.05 compared with pretreatment; **P <0.01 compared with pretreatment.

The glycated hemoglobin levels showed a progressive decline after transplantation, with a maximum decrease 3 months after treatment (baseline, 8.20 ± 1.69%; 1 month, 7.08 ± 1.09%; and 3 months, 6.89 ± 0.90%; *P* <0.01 at 1 month and 3 months compared to the baseline) and were stable at 6.91 ± 0.96% at the 6-month follow-up (p < 0.01) and 7.0 ± 0.60% at the 12-month follow-up (Figure 2A), suggesting a long-lasting effect of WJ-MSC treatment.

### Glycemia

The fasting glucose levels showed a downward trend at different times after intervention, with a maximum decrease 6 months after treatment (baseline, 7.53 ± 2.67 mmol/L; 6 months, 7.12 ± 1.81 mmol/L; *P* <0.05 at 6 months compared to the baseline) and were stable at 7.18 ± 1.8 mmol/L at the 12-month follow-up (*P* <0.05). The oral glucose tolerance test (OGTT) 2 h postprandial showed a maximum reduction 1 month after WJ-MSC transplantation and then slowly rebounded at different time points during the follow-up (baseline, 14.96 ± 4.54 mmol/L; 1 month, 11.43 ± 3.32 mmol/L; 3 months, 12.19 ± 4.24 mmol/L; 6 months, 12.31 ± 3.67 mmol/L; and 12 months 12.25 ± 3.83 mmol/L; *P* <0.05 at 1-, 3-, 6-month and 12-month follow-ups compared to the baseline) (Figure 2B).

### C-peptide levels

**Figure 3:**
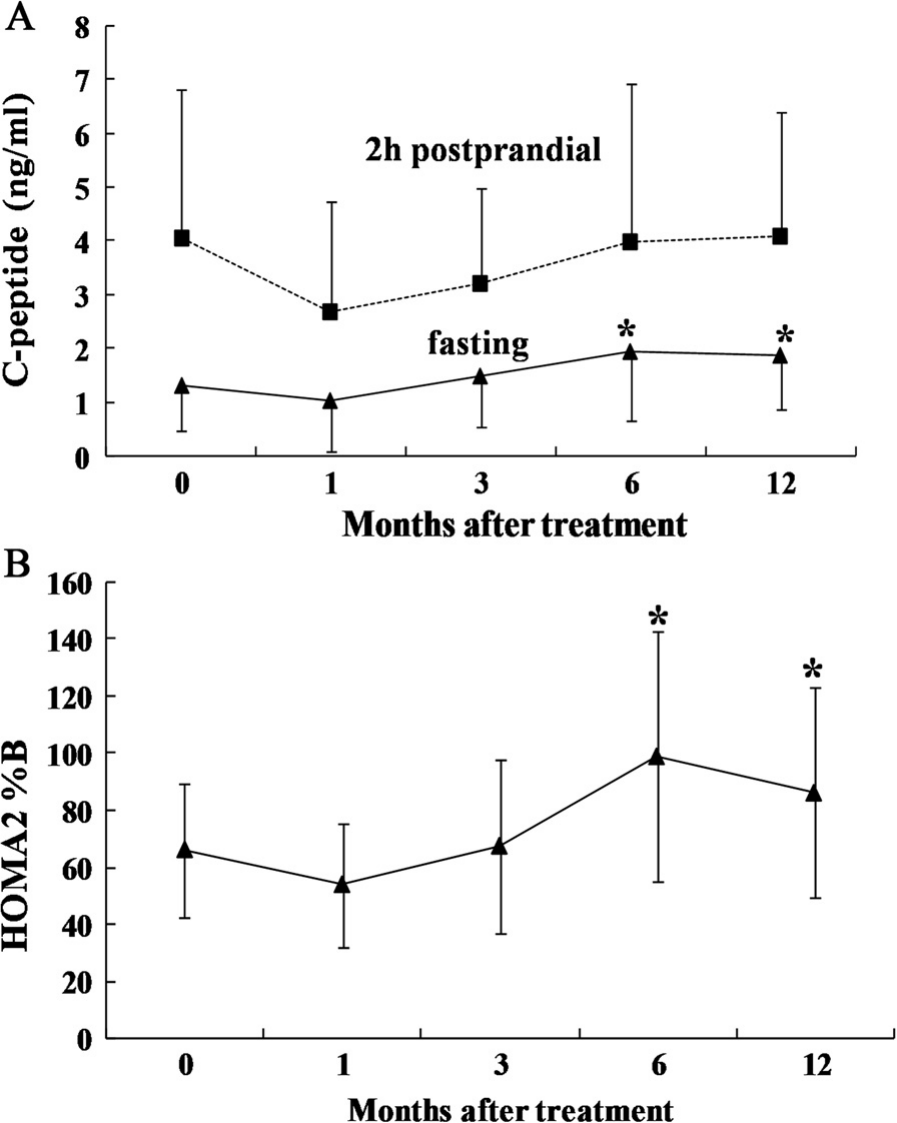
**Changes in C-peptide levels and beta-cell secretory function during the 12-month study period. A**: The changes in fasting C-peptide levels and OGTT 2 h postprandial C-peptide levels between baseline and 1, 3, 6 and 12 months. **B**: Beta-cell secretory function was assessed with HOMA-2B. The results are shown as the mean ± S.D. *P <0.05 compared with pretreatment. HOMA, homeostasis model assessment; OGTT, oral glucose tolerance test.

The fasting serum C-peptide levels first decreased at month 1 and then progressively increased until they reached their peak value at month 6, again slightly decreased at month 12 (baseline, 1.29 ± 0.83 ng/mL; 1 month, 1.03 ± 0.93 ng/mL; 3 months, 1.49 ± 0.94 ng/mL; 6 months, 1.95 ± 1.3 ng/mL; and 12 months, 1.86 ± 1.0 ng/mL; *P* <0.05 at the 6-month and 12-month follow-up compared to the baseline) (Figure 3A). We also detected the OGTT 2 h postprandial C-peptide levels at different times after implantation, but there was no significant difference compared to the baseline during any of the follow-up periods.

### Beta-cell function

The values of fasting glucose and C-peptide in 12/22 patients were within model (HOMA) limits. HOMA-2B increased significantly from 65.99 ± 23.49% at baseline to 98.86 ± 43.91% at the 6-month follow-up (*P* <0.05) and 86.0 ± 37.1% at the 12-month follow-up (Figure 3B), whereas the difference was not statistically significant at the 1-month and 3-month follow-ups.

### Insulin requirement and oral hypoglycemic drugs

**Figure 4:**
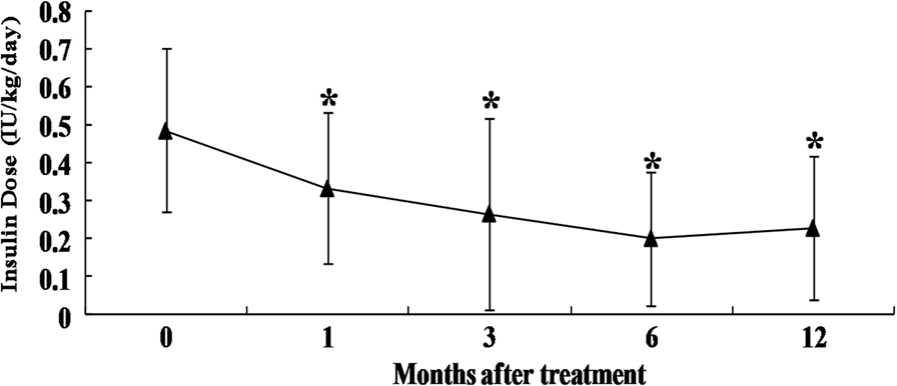
**Changes in daily insulin requirements over time.** This figure shows the insulin requirements of T2DM patients at pre-operation and 1, 3, 6 and 12 months post-transplantation. The results are shown as the mean ± S.D. *P <0.05 compared with pretreatment. The analysis included 17 patients that received exogenous insulin at months 0, 1, 3, 6 and 12. T2DM, Type 2 diabetes mellitus.

After WJ-MSC infusion, the 17 patients who were receiving insulin therapy had a gradual reduction in insulin requirement (baseline, 0.49 ± 0.22 IU/kg/day; 1 month, 0.34 ± 0.20 IU/kg/day; 3 months, 0.27 ± 0.25 IU/kg/day; 6 months, 0.20 ± 0.17 IU/kg/day; and 12 months 0.23 ± 0.19 IU/kg/day; *P* <0.05 at 1-, 3-, 6-month and 12-month follow-up compared to the baseline) (Figure 4). Insulin suspension occurred for 7 of 17 (41%) patients after stem cell transplantation, ranging from two months to six months after treatment (3 ± 1.9 months). These patients remained insulin-free without re-use for a mean time of nine months (9.3 ± 3.8 months) until the last follow-up. In total, 5 of 17 (29%) patients had a reduction in insulin requirement by ≥50%, whereas 1 patient had to resume insulin use at the pretreatment dosage after six months of discontinuation. In the remaining five patients who received insulin treatment, the dosage of insulin decreased to different degrees, except for one patient who was a non-responder. In total, one of five patients who received oral anti-diabetic drugs became completely drug-free three months after treatment without re-use and had good control of blood glucose with only diet and exercise intervention. The time of drug discontinuance was nine months until the last follow-up. The remaining four patients had a reduction of >50% in their oral drug requirement.

### Immunologic tests

**Figure 5:**
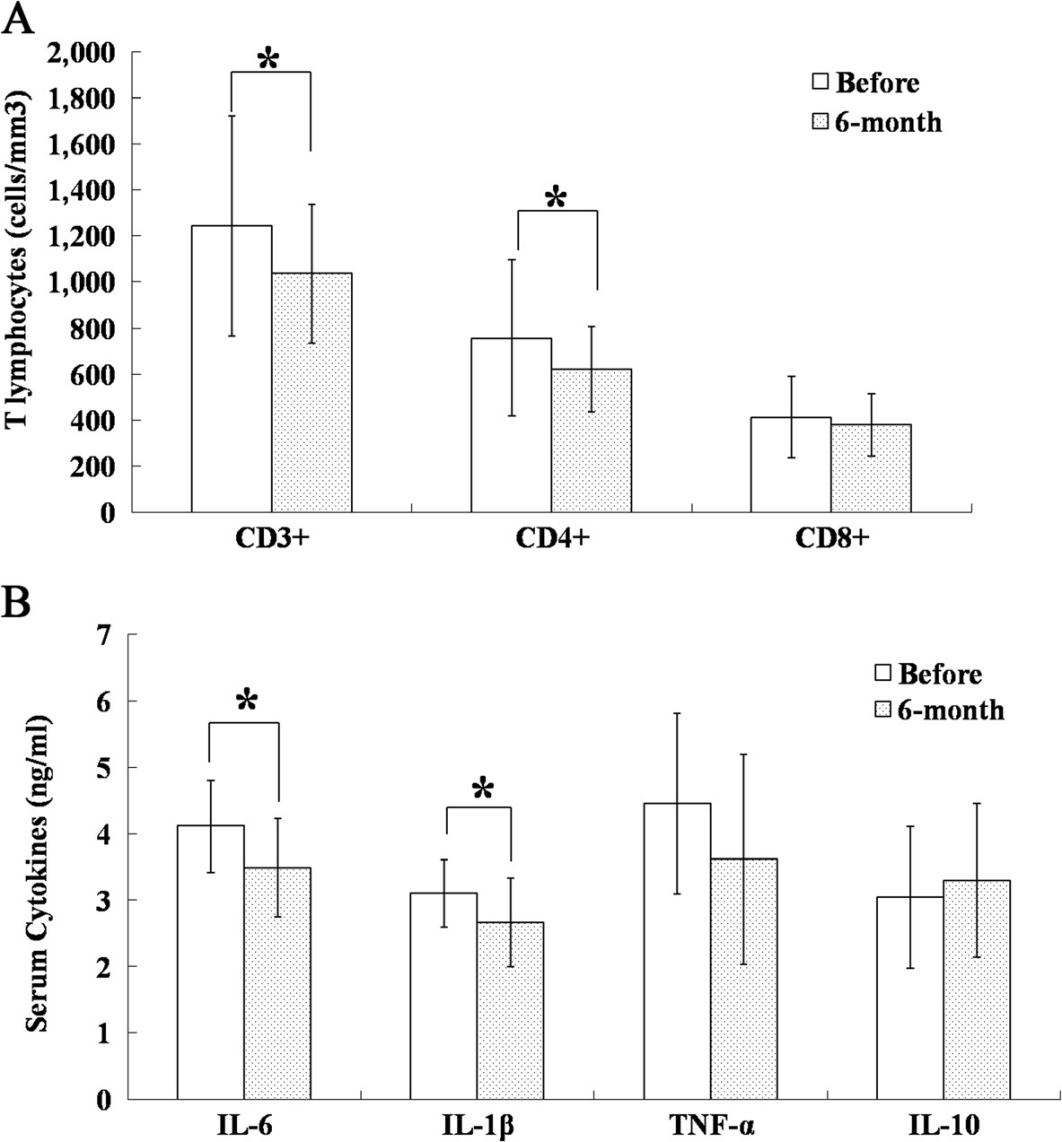
**The immunologic tests and markers of systemic inflammation. A**: The changes in the numbers of CD3^+^, CD4^+^ and CD8^+^ T lymphocytes between baseline and six months. **B**: Changes in the serum levels of IL-6, IL-10, IL-1β and TNF-α between baseline and six months. The results are shown as the mean ± S.D. *P <0.05 compared with pretreatment. The analysis included 22 patients before and six months after umbilical cord mesenchymal stem cell transplantation.

As shown in Figure 5A, there was a decrease in the numbers of T lymphocytes, including CD3^+^, CD4^+^ and CD8^+^ cells, from 1,234 ± 477, 756 ± 339 and 412 ± 177 per cubic millimeter before transplantation to 1,037 ± 300, 621 ± 156 and 379 ± 135 (*P* <0.05 for the CD3^+^ and CD4^+^ T lymphocytes compared to the baseline) per cubic millimeter six months after transplantation, respectively, in all 22 patients who received immunologic tests. The correlation between the change in the levels of fasting C-peptide and the counts of CD3^+^ T lymphocytes was r^2^ = -0.550 (*P* = 0.042). The changes in the numbers of CD4^+^ and CD8^+^ T lymphocytes were not significantly correlated with changes in fasting C-peptide levels.

### Markers of systemic inflammation

We chose six months as the follow-up time to examine the changes in the serum levels of four relevant cytokines: IL-6, IL-10, IL-1β and TNF-α. We found that the serum levels of interleukin-6 (*P* <0.05) and interleukin-1β (*P* <0.05) were significantly reduced six months after WJ-MSC transplantation compared with pretreatment in all 22 patients. The serum levels of TNF-α were also substantially decreased six months after WJ-MSC treatment (*P* >0.05), and IL-10 levels were up-regulated at the same visit (*P* >0.05) (Figure 5B). The correlation between the change in levels of fasting C-peptide and IL-6 was r^2^ = -0.766 (*P* = 0.001). The changes in IL-1β, TNF-α and IL-10 were not significantly correlated with changes in fasting C-peptide levels.

### Adverse events

The side effects of WJ-MSC transplantation included mild and moderate fever in 3 of the 22 patients, which generally occurred on the first operative day and spontaneously returned to normal levels; subcutaneous hematoma at the injection site in one case on Day 1 after the arterial intervention operation (which resolved after seven days); and nausea, vomiting and headache in one patient, who recovered spontaneously within one week. Data suggest a temporary decrease in levels of C-peptide and beta-cell function at one month after treatment, possibly related to intrapancreatic endovascular injection. The safety of intrapancreatic endovascular delivery is a concern and deserves further investigation. All 22 patients are still under follow-up for possible late-onset side effects of the WJ-MSC treatment.

## Discussion

In the present study, we document, for the first time, that WJ-MSC transplantation decreased the level of HbA1c; increased the level of fasting C-peptide; decreased the fasting glucose level, 2 h postprandial blood glucose level, insulin requirement and oral hypoglycemic drugs; and reduced the systemic inflammation and T lymphocyte counts in patients with T2DM.

HbA1c is a glycemic indicator and reflects the average blood glucose over the preceding 8 to 12 weeks [17]. We detected the maximum decrease in HbA1c in the first three months after WJ-MSC transplantation, and the maximum decrease in both the fasting glucose levels and 2 h postprandial glucose levels occurred at the same time, implying that WJ-MSC treatment can improve glycemic control in a short time. It is noteworthy that the levels of the fasting serum C-peptide and 2 h postprandial C-peptide decreased to different degrees in this period. Strangely, we also found that β cell function decreased, as assessed with the HOMA calculator, in 12 of 22 patients at one month. The intrapancreatic endovascular injection could cause impairment of the organ in case one or more of the following events were to occur: 1) puncturing a large artery causing a major bleed; 2) causing an instant blood-mediated inflammatory reaction (IBMIR) in the pancreatic circulation and 3) causing the formation of blood clots, all of which could ultimately lead to a pancreatic infarction, and erase precious beta cells. The use of a contrast agent during the intervention may facilitate the identification of potential injuries to the pancreas. Further investigations are needed to address the safety concerns of this delivery method. At this time-point, 17 patients who received insulin therapy had a reduction in their insulin requirement from 0.48 ± 0.21 IU/kg/day at baseline to 0.34 ± 0.20 IU/kg/day. The most probable reason for this contradictory phenomenon is that the infused WJ-MSCs rapidly improved general insulin resistance, which led to a reduction in endogenous insulin secretion and the need for exogenous insulin injection. At the subsequent three-month and six-month follow-ups, the levels of fasting C-peptide increased gradually and reached a peak value at six months, which suggests that WJ-MSC transplantation enhanced the basal insulin secretion. The results of β cell function, as assessed with the HOMA calculator, in 12 out of 22 patients also verified this conclusion. At this stage, the fasting glucose levels showed a downward trend but were not statistically significant. In addition, the 2 h postprandial glucose levels showed a significant decrease compared to the baseline and remained constant, suggesting a longer lasting effect of WJ-MSC treatment.

Because the HbA1c and C-peptide of patients who received WJ-MSC treatment showed the best control at the sixth month, we chose the sixth month to examine the therapeutic mechanism of WJ-MSC in 22 enrolled patients. Mounting evidence has demonstrated that chronic and low-grade inflammation plays an important role in the development and progression of T2DM [18]. Inflammation can not only impair insulin signaling and contribute to insulin resistance [19,20] but can also trigger β cell apoptosis and reduce insulin secretion [8]. Many pro-inflammatory cytokines, such as TNF-α, IL-6 and IL-1β, have been demonstrated to be elevated in T2DM patients and participate in the development of insulin resistance [21,22]. Numerous studies have verified that MSCs show promising therapeutic potential in autoimmune disease [11] and neurological abnormalities [23] through the modulation of the inflammatory condition. Our studies also demonstrated that the levels of pro-inflammatory cytokines, including IL-6 and IL-1β, were significantly decreased six months after WJ-MSC transplantation. CM Larsen and colleagues found that an IL-1 receptor antagonist improved glycemia and beta-cell secretory function accompanied with a reduction in markers of systemic inflammation, including C-reactive protein and IL-6. Through correlation analysis, the serum pro-inflammatory cytokines were not significantly correlated with the improvement in HbA1c [24]. Our data also did not show a correlation among the levels of HbA1c and IL-1β and IL-6, but we found a significant correlation between the changes in the levels of fasting C-peptide and IL-6, suggesting that reduced systemic inflammation may play an important role in improving basal insulin secretion. We believe that WJ-MSCs may exert their therapeutic potential partly by regulating systemic inflammation.

Shawn Winer *et al*. found that CD4^+^ T lymphocytes residing in visceral adipose tissue controlled insulin resistance in mice with diet-induced obesity, and a similar process occurred in humans. The treatment of obese wild type (WT) and ob/ob (leptin-deficient) mice with a CD3-specific antibody or its F(ab’)_2_ fragment reduced the predominance of Th1 cells compared to Foxp3+ cells and reversed insulin resistance for months [25]. Ulrich Kintscher *et al*. showed that pro-inflammatory T-lymphocytes played an important role in the initiation and perpetuation of adipose tissue inflammation and the development of insulin resistance (IR) [26]. Our data showed that the numbers of CD3^+^ and CD4^+^ T lymphocytes were reduced in 12 of 14 patients three months after WJ-MSC treatment, and there was a significant correlation between the changes in the levels of fasting C-peptide and the numbers of CD3^+^ T lymphocytes, suggesting that WJ-MSC transplantation may participate in the regulation of the immune process and reduce inflammation and insulin resistance, improving diabetic symptoms. Future studies are needed to determine the correlation between the T lymphocyte counts and diabetic symptoms in more cases.

For MSC delivery, some studies have chosen intravenous delivery [27] or endovascular catheterization [4], and a few studies have used both approaches. We believe that the majority of intravenously transplanted MSCs will be trafficked to the lung and the other peripheral tissues [28]; thus, the number of MSCs recruited to islets is limited. Local intra-arterial (IA) injections can enhance the accumulation and increase the number of MSCs in islets. Nevertheless, safety concerns related to this delivery method will have to be addressed in future studies. T2DM is a metabolic disease characterized by insulin resistance and beta-cell dysfunction. Therefore, we believe that all of the delivery approaches have limitations, and we chose a combination of two approaches in our study. MSCs injected through intravenous delivery improve the symptoms of peripheral tissues, and IA injected MSCs may exert functions in islets. Based on our study results, we speculate that intravenously infused MSCs can inhibit systemic inflammation and improve insulin resistance, whereas local intra-arterial injection infused MSCs can improve islet inflammation and reduce the damage to β cells.

Many studies have reported a therapeutic effect of umbilical cord-derived MSCs in a variety of diseases and no obvious adverse effects were reported [11,29,30]. XY XY and colleagues designed a clinical study to treat foot disease in patients with type 2 diabetes mellitus using hUCB-MSCs and there is no related adverse effect reported [6]. J Hu *et al*. assessed the long-term effects of the implantation of WJ-MSCs for new-onset T1DM. There were no obviously adverse reactions in any of the patients who completed the study protocol, and no chronic side effects or lingering effects appeared during the follow-up [29]. Lingyun Sun’ study explored the therapeutic effect of UC-MSCs in severe and treatment-refractory systemic lupus erythematosus (SLE). No treatment-related adverse events occurred during or after UC-MSC transplantation, and UC-MSC transplantation was well tolerated by all patients [11]. We also have the same results on the safety of WJ-MSC transplantation with all the above trials.

## Conclusions

These findings suggest that WJ-MSC transplantation may have a possible therapeutic potential in T2DM. Furthermore, we showed that the therapeutic effect was associated with an improvement in β cell function, systemic inflammation and/or immunological regulation. Further follow-up and large-scale placebo-controlled clinical studies are necessary to fully elucidate the role of WJ-MSCs in the treatment of T2DM.

## Abbreviations

DMEM: Dulbecco’s modified Eagle’s medium; FBG: Fasting blood glucose; HbA1c: Glycated hemoglobin; HOMA: Homeostasis model assessment; hUCB-MSC: Human umbilical cord blood mesenchymal stem cells; IA: Intra-arterial; IR: Insulin resistance; OGTT: Oral glucose tolerance test; PBG: Post-meal blood glucose; T2DM: Type 2 diabetes mellitus; WJ-MSCs: Umbilical Cord Wharton’s Jelly-derived mesenchymal stem cells.

## Competing interests

The authors declare that they have no competing interests.

## Authors’ contributions

YA, XL and PZ contributed to the conception and design, collection and assembly of data, data analysis and interpretation, manuscript writing, and final approval of the manuscript. XW, GD and ZZ contributed to acquisition of data, prepared the stem cells and performed laboratory experiments. RH, HC, XN and JS contributed to analysis and interpretation of the data and final approval of the manuscript. All authors read and approved the final manuscript.
